# LncRNA AGAP2-AS1 augments cell viability and mobility, and confers gemcitabine resistance by inhibiting miR-497 in colorectal cancer

**DOI:** 10.18632/aging.102940

**Published:** 2020-03-23

**Authors:** Sen Hong, Zhenkun Yan, YuMei Song, MiaoMiao Bi, Shiquan Li

**Affiliations:** 1 Department of Colorectal and Anal Surgery, The First Hospital of Jilin University, Changchun 130021, Jilin Province, China; 2 Department of Endoscopy Center, China-Japan Union Hospital of Jilin University, Changchun 130033, Jilin Province, China; 3 Department of Thoracic Oncology, Tumor Hospital of Jilin Province, Changchun 130000, Jilin Province, China; 4 Department of Ophthalmology, The China-Japan Union Hospital of Jilin University, Jilin University, Changchun 130033, Jilin Province, China

**Keywords:** long non-coding RNAs (lncRNAs), colorectal cancer (CRC), miR-497, fibroblast growth factor receptor 1 (FGFR1), survival

## Abstract

Background: Most recently, long non-coding RNAs (lncRNAs) emerge as crucial modulators in many biological processes, such as embryonic development, cell growth, and tumorigenesis. However, the correlations between lncRNAs and colorectal cancer (CRC) cell proliferation, metastasis, and gemcitabine resistance are not well understood.

Results: The expression of AGAP2-AS1 was overexpressed in CRC tissues and negatively correlated with the survival of patients with CRC. AGAP2-AS1 promoted CRC cell proliferation and inhibited apoptosis. Moreover, AGAP2-AS1 enhanced the chemoresistance of CRC cells to gemcitabine. In addition, AGAP2-AS1 enhanced the migration and invasion of CRC cells. Mechanistic studies showed that AGAP2-AS1 regulated fibroblast growth factor receptor 1 (FGFR1) expression by sponging miR-497 in CRC progression.

Conclusion: We identified an oncogenic role of AGAP2-AS1 in the development and progression of CRC.

Methods: qRT-PCR was used to measure the expression of AGAP2 Antisense RNA 1 (AGAP2-AS1) in 116 cases of CRC and adjacent normal tissues. Luciferase reporter assays was used to detect the interaction between AGAP2-AS1 and miR-497. The xenograft tumor experiment was used to study the in vivo function of AGAP2-AS1.

## INTRODUCTION

Colorectal cancer (CRC) ranks as the third-highest malignant tumors around the world [1]. Changes in genetic and epigenetic levels of oncogenes or tumor suppressors are supposed to be important factors in CRC development and progression; however, the genetic and epigenetic basis of CRC remains unclear. Growing evidence has shown that long non-coding RNAs (lncRNAs) are implicated in the tumorigenesis and progression of CRC [2].

LncRNAs are a class of transcripts with more than 200 nucleotides and do not have protein coding ability [3, 4]. Recent studies have shown that lncRNAs play vital roles in regulating chromosome inactivation and tumorigenesis [5]. AGAP2-AS1 is transcribed by a gene located at 12q14.1 with a length of 1567 nt and abnormally expressed in various human cancers [6]. AGAP2-AS1 has been shown to play a carcinogenic role in hepatocellular carcinoma, glioblastoma, and pancreatic cancer [7–9]. In non-small cell lung cancer, lncRNA AGAP2-AS1 acts as an oncogene that inhibits the expression of large tumor suppressor kinase 2 (LATS2) and Kruppel-like factor 2 (KLF2) via interacting with EZH2 and LSD1 [6]. It has been shown that lncRNA AGAP2-AS1 promotes the proliferation and invasion of gastric cancer cells [10]. Drug resistance remains a big challenge in effective treatment. Increased AGAP2-AS1 expression has been shown to increase chemoresistance of breast cancer to trastuzumab through epigenetic regulation of MyD88 [11]. On the other hand, Zheng et al. reported that AGAP2-AS1 enhances trastuzumab resistance of breast cancer cells by packaging into exosomes [12]. However, the significance of AGAP2-AS1 in the progression and drug resistance in CRC remains unclear.

In this study, we explored the expression of AGAP2-AS1 in CRC and analyzed its association with the survival of CRC patients. Furthermore, functional analysis and mechanistic studies were performed to determine the important role of AGAP2-AS1 in tumorigenesis and chemoresistance in CRC.

## RESULTS

### AGAP2-AS1 expression was associated with survival in patients with CRC

**Figure 1:**
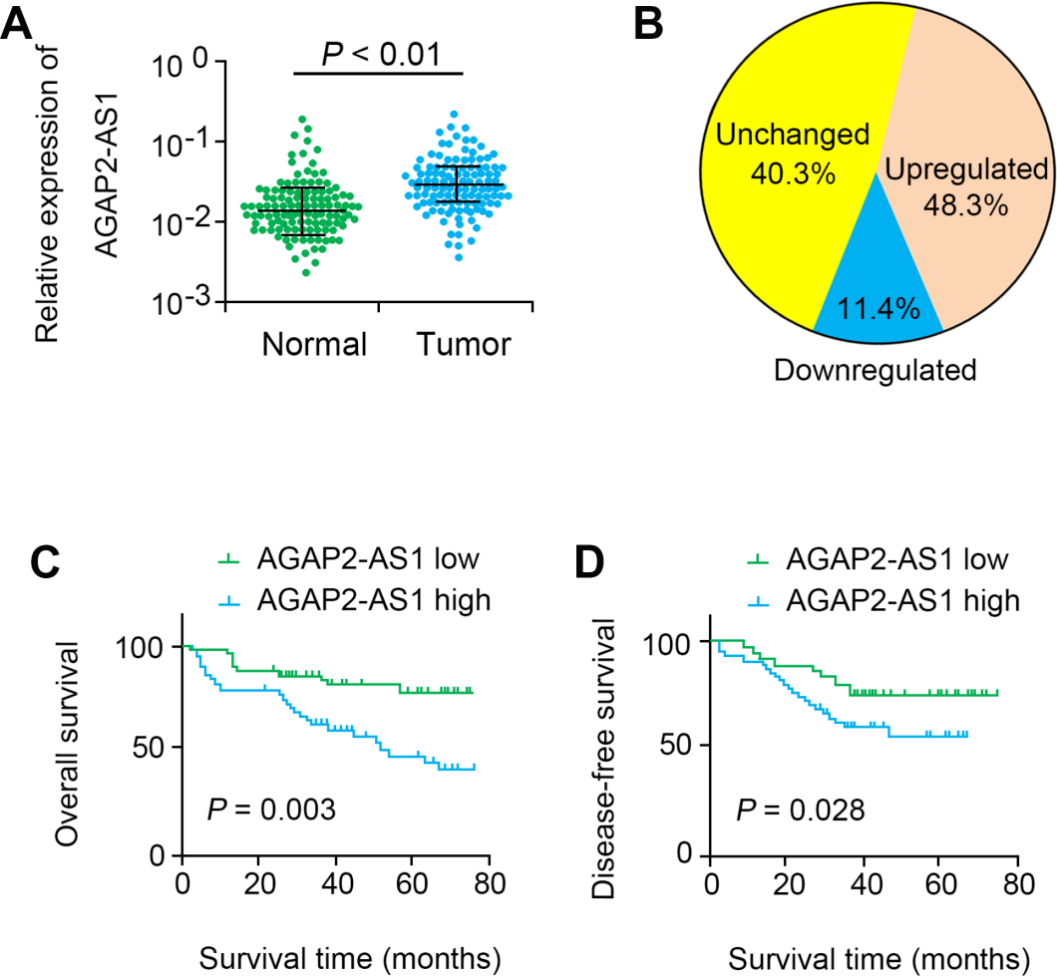
**AGAP2-AS1 was overexpressed in CRC tissues.** (**A**) Relative expression levels of AGAP2-AS1 in 116 paired CRC tissues and paired noncancerous tissues were quantified by qRT-PCR. (**B**) AGAP2-AS1 was upregulated (> 2-fold) in 48.3% of the CRC tissues. (**C**, **D**) Kaplan–Meier survival analysis of the OS and DFS in CRC patients with low or high AGAP2-AS1 expression.

Our data indicated that AGAP2-AS1 was significantly up-regulated in CRC tissues (P < 0.001, Figure 1A). In 48.3% (56 of 116) CRC tissues, AGAP2-AS1 was significantly up-regulated compared with non-cancerous tissues (> 2 folds, Figure 1B). Moreover, the level of AGAP2-AS1 was highly correlated to tumor stage (P=0.016, Table 1). Additionally, survival analysis showed that the OS (log rank = 8.982, P = 0.003) and DFS (log rank = 5.017, P = 0.028) in the AGAP2-AS1-high expression group were remarkably shorter than those in the AGAP2-AS1-low expression group (Figure 1C, 1D). Multivariate analysis further showed that AGAP2-AS1 was an independent prognostic factor for CRC (HR=2.405, 95% CI=1.137–6.023, P=0.023, Table 2).

**Table 1:** Correlation of the expression of AGAP2-AS1 with clinicopathologic features.

**Characteristics**	**AGAP2-AS1**		**P-value**
	**Low**	**High**	
Ages (years)			
< 60	33	31	1.681
≥ 60	25	28	
Gender			
Male	32	35	0.472
Female	26	23	
Tumor size (cm)			
< 5	43	42	0.615
≥ 5	15	16	
Location			
Colon	31	32	0.850
Rectum	27	26	
Differentiation			
Well/moderately	49	46	0.313
Poorly	9	12	
Tumor stage			
I /II	34	17	0.016
III /IV	24	39	
Depth of tumor			
T1/ T2	15	10	0.087
T3/T4	43	48	
Distant metastasis			
Absent	48	49	0.634
Present	10	9	

**Table 2:** Univariate and multivariate regression analyses associated with prognosis in CRC patients.

**Characteristics**	**Subset**	**Univariate analysis**		**Multivariate analysis**	
		**P-value**	**HR (95% CI)**	**P-value**	**HR (95% CI)**
Ages (years)	< 60/≥ 60	0.437	0.817 (0.387–1.608)	-	-
Gender	Male/female	0.905	0.921 (0.494–1.864)	-	-
Tumor size	< 5 cm/ ≥ 5 cm	0.372	1.295 (0.702–2.853)	-	-
Location	Colon/rectum	0.974	2.130 (0.534–2.105)	-	-
Depth of tumor	T1 + T2/T3 + T4	0.028	8.627 (1.148–60.113)	0.237	4.083 (0.549–30.370)
Differentiation	Well + moderately/poorly	0.013	1.468 (1.203–5.461)	0.083	2.031 (0.871–5.617)
Tumor stage	I + II/III + IV	0.000	9.855 (4.021–28.654)	0.001	8.046 (1.468–23.656)
Distant metastasis	Present/absent	0.426	0.713 (0.191–1.750)	-	-
AGAP2-AS1	High/low	0.001	3.774 (1.652–8.382)	0.023	2.405 (1.137–6.023)

### AGAP2-AS1 promoted the proliferation of CRC cells

**Figure 2:**
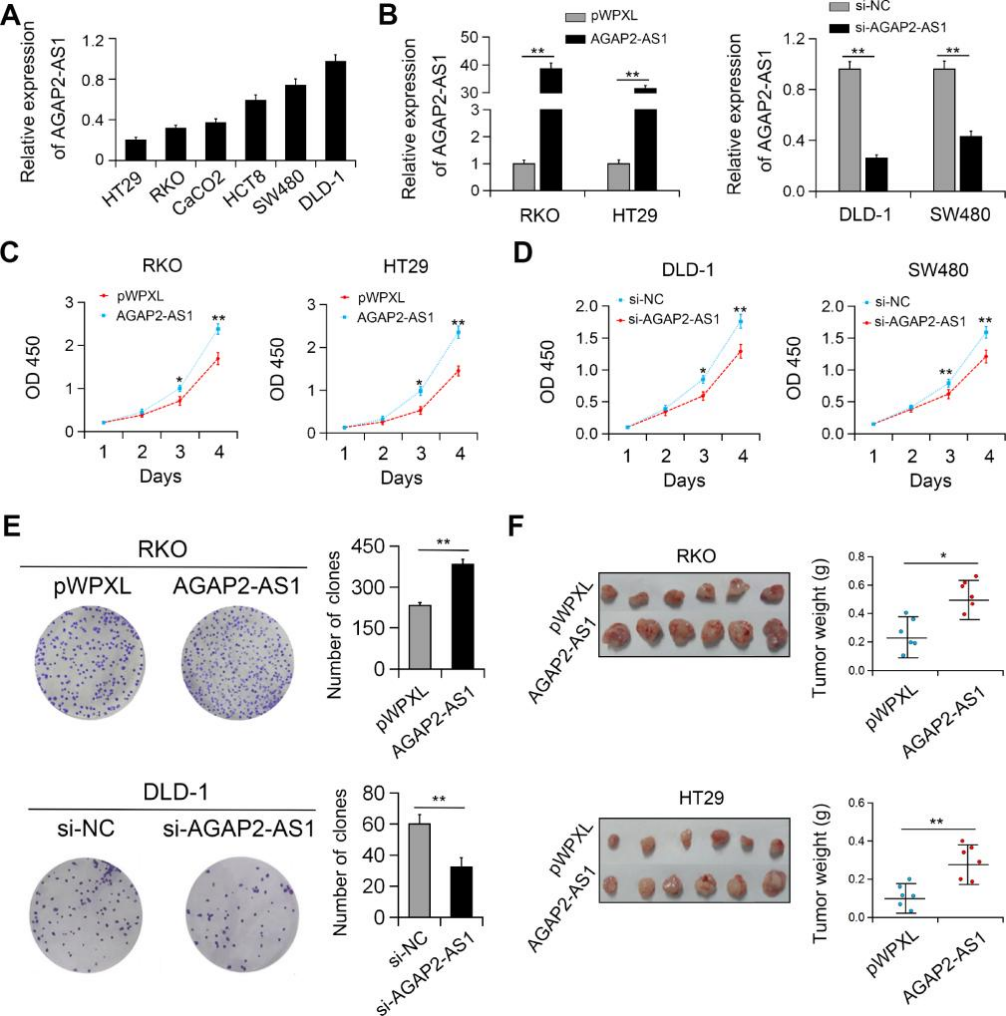
**AGAP2-AS1 promoted cell viability both in vitro and in vivo.** (**A**) Relative expression of AGAP2-AS1 in CRC cell lines. (**B**) Validation of overexpression and knockdown efficacy of AGAP2-AS1 in CRC cell lines by qRT-PCR. (**C**, **D**) Effects of AGAP2-AS1 overexpression and downregulation on CRC cell proliferation were measured by CCK-8 assay. (**E**) Effects of AGAP2-AS1 on colony formation in CRC cells. (**F**) AGAP2-AS1 overexpression promoted CRC tumorigenesis in a xenograft mouse model. *P < 0.05; **P < 0.01.

In vitro studies showed that the expression of AGAP2-AS1 was relatively higher in DLD-1 and SW480 CRC cells and relatively lower in RKO and HT29 cells (Figure 2A). Therefore, gain (DLD-1 and SW480 cells) and loss (RKO and HT29 cells) of function studies of AGAP2-AS1 were performed in these cells, respectively (Figure 2B). We identified that overexpression of AGAP2-AS1 significantly promoted the viability and colony formation of CRC cells. On the contrary, AGAP2-AS1 silencing reduced cell proliferation and colony forming ability (Figure 2C–2E). In agreement with these results, in vivo studies showed that ectopic expression of AGAP2-AS1 promoted the growth of RKO and HT29 cells in nude mice (Figure 2F).

### AGAP2-AS1 silencing induced G1/M phase cell cycle arrest and increased gemcitabine sensitivity to CRC cells

**Figure 3:**
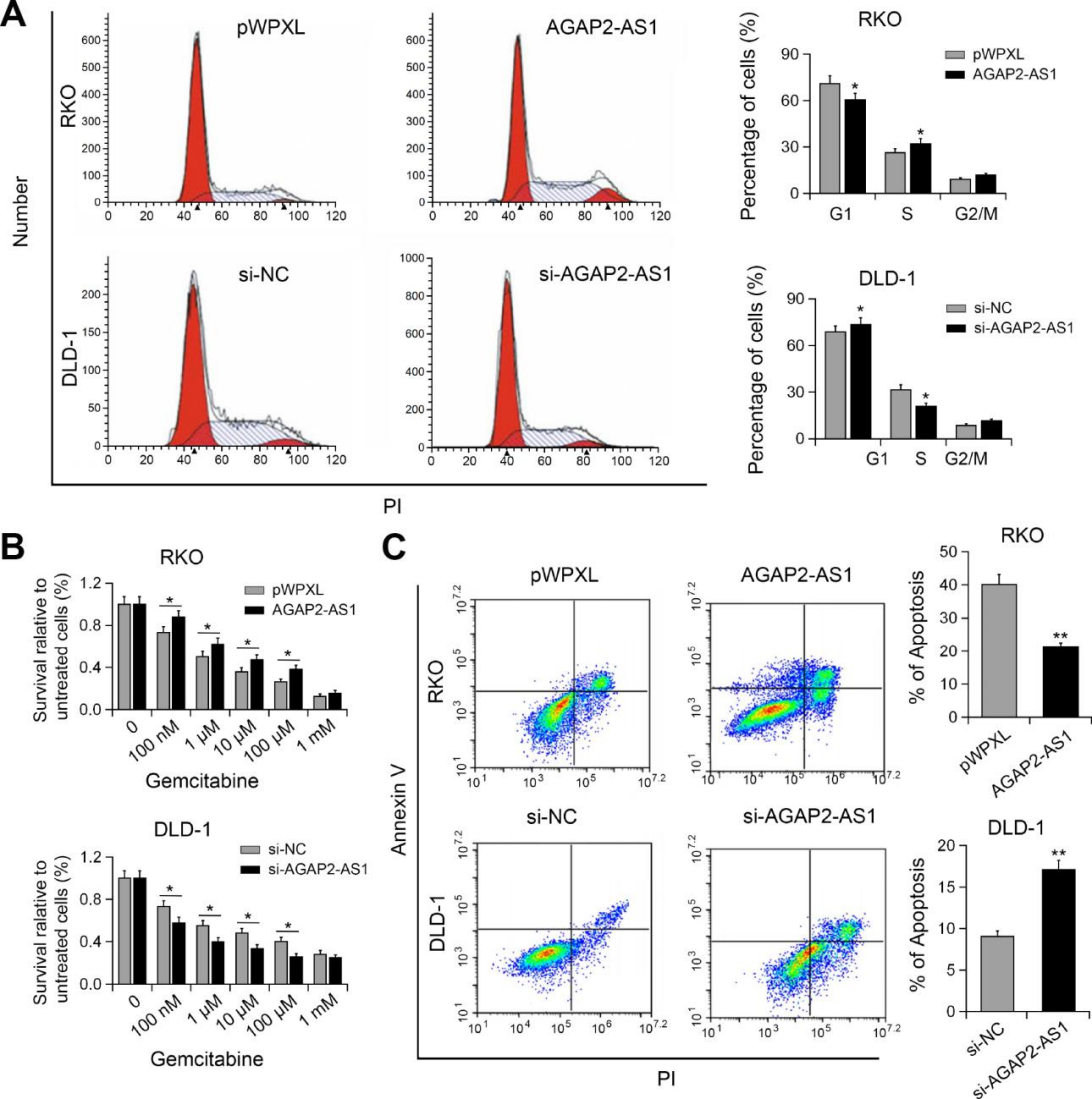
**AGAP2-AS1 promoted cell cycle progression and conferred gemcitabine resistance.** (**A**) Cell cycle in RKO cells transfected with pWPXL-AGAP2-AS1 or pWPXL, and DLD-1 cells transfected with si-AGAP2-AS1 or si-NC were analyzed. (**B**) The sensitivity of CRC cells to gemcitabine were decreased by AGAP2-AS1. (**C**) Apoptosis of CRC cells after AGAP2-AS1 overexpression or knockdown in the presence of gemcitabine. *P < 0.05; **P < 0.01.

To understand the mechanism underlying AGAP2-AS1-mediated cell proliferation, we performed cell cycle analysis. As shown in Figure 3A, knockdown of AGAP2-AS1 resulted in increased cell proportion in G1/M phase, whereas overexpression of AGAP2-AS1 promoted the cell cycle progression. Gemcitabine has been used in the treatment of metastatic CRC [13]. Gemcitabine resistant using CRC cell model has also been reported [14]. Therefore, we investigated whether AGAP2-AS1 plays a role in the anti-cancer activity of gemcitabine. Interestingly, CCK-8 assay showed that overexpression of AGAP2-AS1 increased the resistance of RKO cells to gemcitabine, while silencing of AGAP2-AS1 enhanced the sensitivity of DLD-1 cells to gemcitabine (Figure 3B). Moreover, overexpression of AGAP2-AS1 antagonized gemcitabine-induced apoptosis, whereas AGAP2-AS1 knockdown promoted gemcitabine-induced apoptosis (Figure 3C). These results suggest that AGAP2-AS1 modulated gemcitabine resistance to CRC cells.

### AGAP2-AS1 promoted mobility of CRC cells

**Figure 4:**
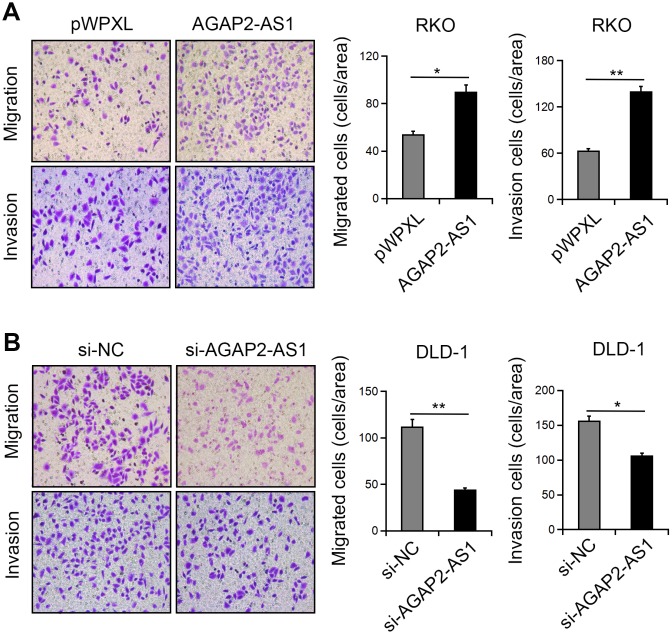
**AGAP2-AS1 promoted the mobility in CRC cells.** (**A**, **B**) Representative images of cell migration and invasion after AGAP2-AS1 overexpression or knockdown. *P < 0.05; **P < 0.01.

Next, we investigated the function of AGAP2-AS1 in CRC cell migration and invasion. Transwell assay showed that overexpression of AGAP2-AS1greatly enhanced the migration and invasion of RKO cells. In contrast, knockdown of AGAP2-AS1 inhibited the numbers of migrated and invaded DLD-1 cells (Figure 4A, 4B). These results suggest that AGAP2-AS1 has a promoting effect on CRC cell motility.

### AGAP2-AS1 acted as a sponge for miR-497

**Figure 5:**
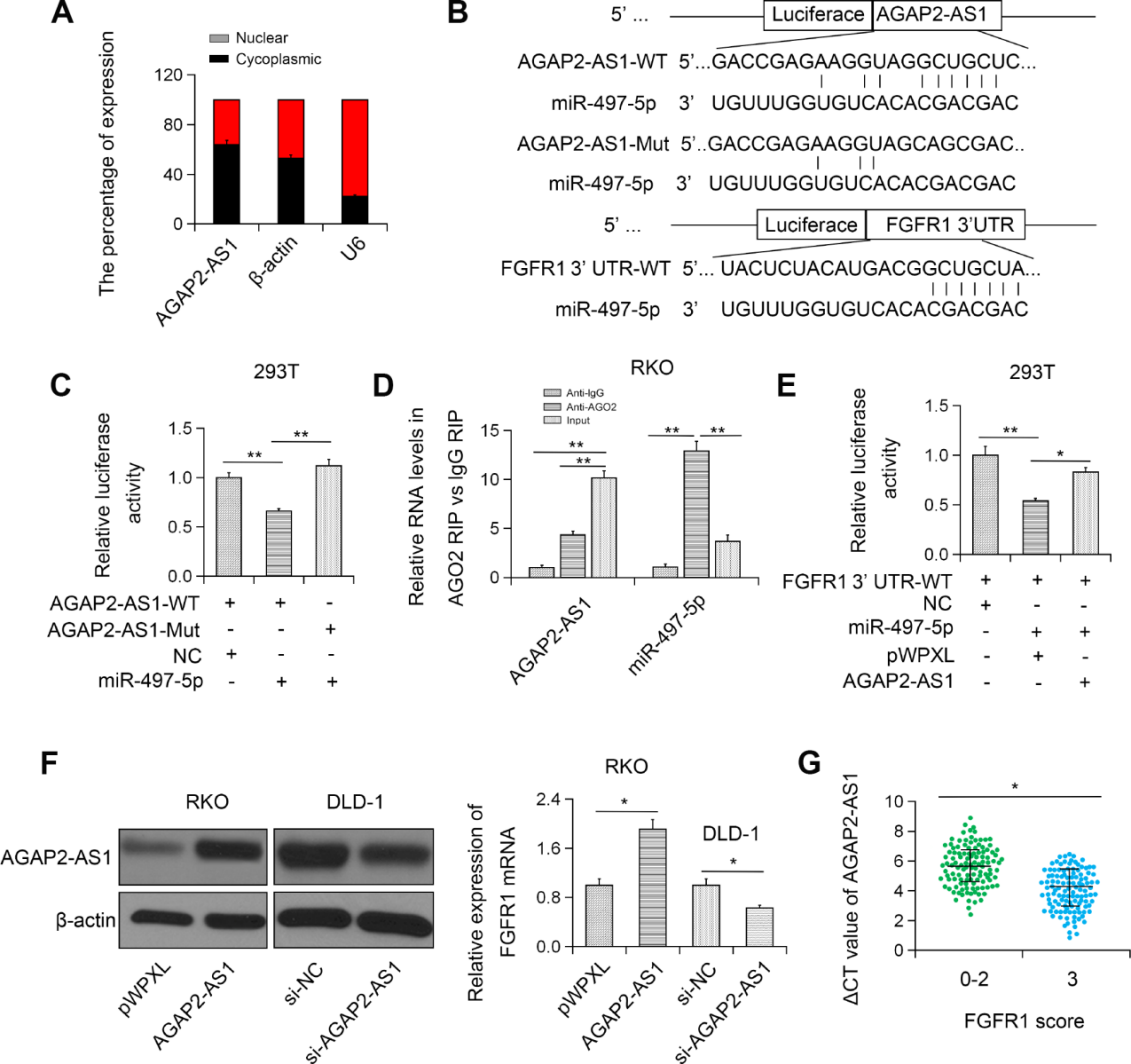
**AGAP2-AS1 sponged miR-497 and modulated FGFR1 expression.** (**A**) Subcellular localization of AGAP2-AS1 was detected in RKO cell line. (**B**) miR-497-binding sequence in AGAP2-AS1 and FGFR1 3'UTR. (**C**) Luciferase activity of pLuc-AGAP2-AS1 WT or Mut co-transfected with miR-497. (**D**) Cellular lysates from RKO cells were used for RIP with an anti-Ago2 antibody or IgG antibody. (**E**) miR-497 and pLuc-FGFR1 3'UTR were co-transfected with pWPXL-AGAP2-AS1 into 293T cells. (**F**) The expression level of FGFR1 in RKO cells transfected with pWPXL-AGAP2-AS1 and in DLD-1 cells transfected with si-AGAP2-AS1. (**G**) Correlation between FGFR1 and AGAP2-AS1 expression. *P < 0.05; **P < 0.01.

To further investigate the mechanism of AGAP2-AS1 in CRC, we determined the subcellular localization of AGAP2-AS1. We found that AGAP2-AS1 was primarily localized in the cytoplasm (Figure 5A). Based on bioinformatics analysis (TargetScan and miRanda), we found that AGAP2-AS1 could target miR-497 (Figure 5B). To access the direct interaction between AGAP2-AS1 and miR-497, pLuc-AGAP2-AS1 vectors with WT/Mut miR-497 binding sequences were constructed and a luciferase reporter assay was performed. The result showed that transfection with miR-497 significantly reduced the luciferase activity of pLuc-AGAP2-AS1-WT, but had no significant effect on pLuc-AGAP2-AS1-Mut (Figure 5C). Furthermore, RIP analysis data confirmed the enrichment of both AGAP2-AS1 and miR-497 in Ago2 complex, indicating that AGAP2-AS1 could directly bind to miR-497 (Figure 5D). These results suggest that AGAP2-AS1 interacts with miR-497.

### FGFR1 was targeted by miR-497 in CRC cells

Furthermore, dual luciferase reporter assay showed that overexpression of AGAP2-AS1 blocked the inhibition of the luciferase activity of Fibroblast growth factor receptor 1 (FGFR)-3’UTR mediated by miR-497 (Figure 5E). Moreover, FGFR1 expression was greatly enhanced in CRC cells overexpressing AGAP2-AS1 (Figure 5F). On the contrary, silencing of AGAP2-AS1 remarkably restrained the expression of FGFR1 in RKO and DLD-1 CRC cell lines (Figure 5F). Furthermore, FGFR1 was found to be positively correlated with AGAP2-AS1 in CRC tissues (Figure 5G). Together, these results indicate that AGAP2-AS1 can modulate FGFR1 by sponging miR-497.

### AGAP2-AS1 played a carcinogenic role by modulating miR497/FGFR1 axis

**Figure 6:**
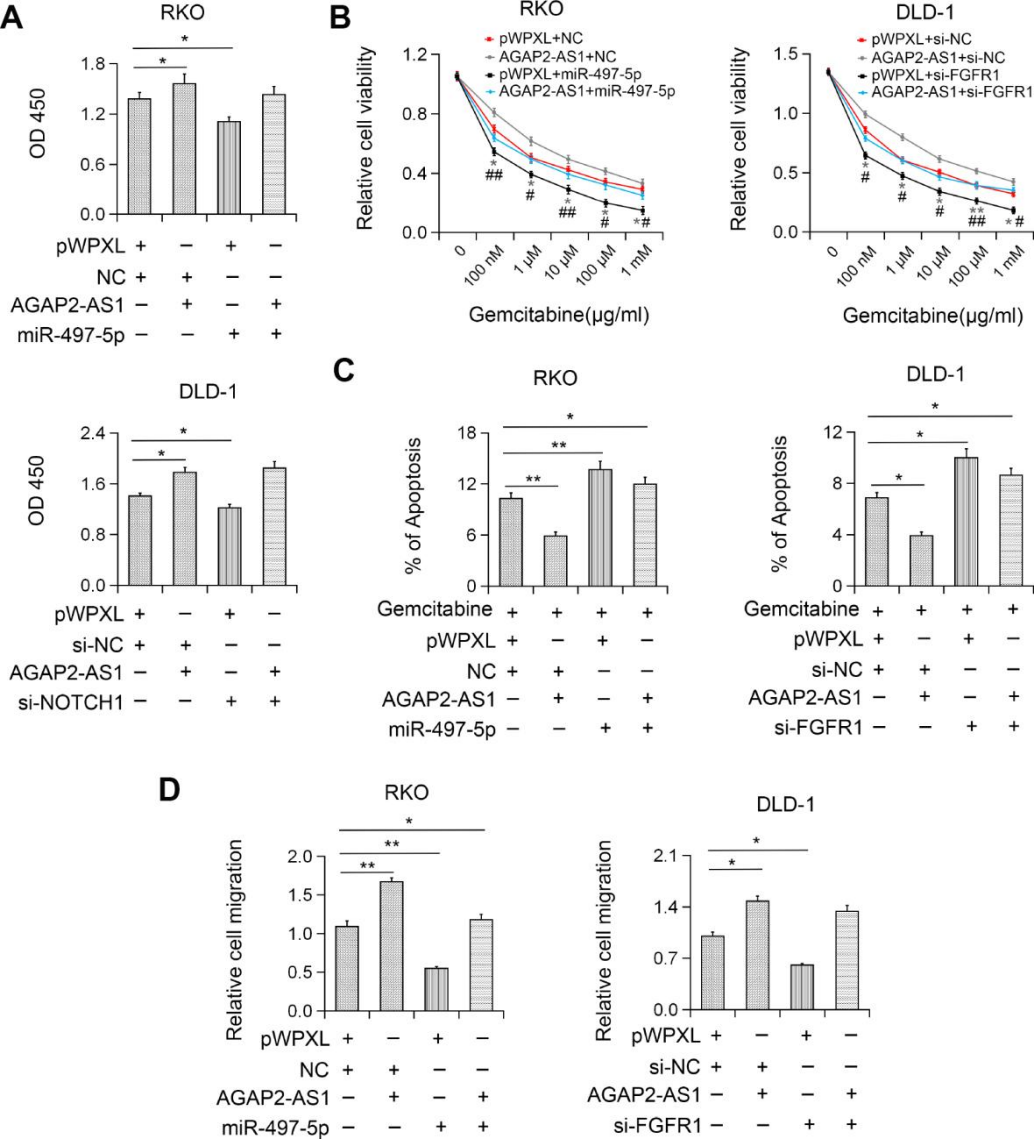
**AGAP2-AS1 exerted carcinogenic function in CRC by regulating the miR-497/FGFR1 axis.** (**A**) Increased cell viability induced by AGAP2-AS1 overexpression was abolished after miR-497 overexpression or FGFR1 knockdown by CCK-8 assay. (**B**) Increased gemcitabine resistance in CRC cells after pWPXL-AGAP2-AS1 transfection was abolished by miR-497 overexpression or FGFR1 knockdown. (**C**) AGAP2-AS1 overexpression suppressed gemcitabine-induced apoptosis and this effect was partially inhibited by miR-497 overexpression or FGFR1 knockdown. (**D**) miR-497 overexpression or FGFR1 knockdown blocked CRC cell migration induced by AGAP2-AS1. *P < 0.05; **P < 0.01.

To explore whether AGAP2-AS1 plays an oncogenic role in CRC by modulating the miR-497/FGFR1 axis, the effects of miR-497 or FGFR1 on AGAP2-AS1- induced cell proliferation and migration were investigated. We found that miR-497 overexpression or FGFR1 knockdown inhibited AGAP2-AS1-induced CRC cell proliferation (Figure 6A). Also, miR-497 overexpression or FGFR1 knockout significantly reversed the gemcitabine resistance induced by AGAP2-AS1 (Figure 6C). Moreover, after miR-497 overexpression or FGFR1 knockdown, the migration ability of AGAP2-AS1-overexpressing CRC cells was reversed (Figure 6D), indicating that AGAP2-AS1 played a carcinogenic role by sponging miR-497 and upregulation of FGFR1 expression in CRC.

## DISCUSSION

Accumulated evidence has revealed different regulatory roles of lncRNAs in human disease, particularly in the development and progression of tumors [15]. Chemotherapy resistance and recurrence of tumors are common in cancers, which pose a critical issue for clinicians [16]. Dysregulation of LncRNAs is often found in cancer and associated with chemotherapy resistance [17]. For example, long non-coding RNA UCA1 sensibilize cisplatin/gemcitabine resistance by regulation of miR-196a-5p in bladder cancer cell [18]. In pancreatic cancer, the LncRNA HOTTIP promoted gemcitabine resistance through modulating HOXA13 [19]. LncRNA PVT1 targets miR-152 and enhances the resistance of osteosarcoma to gemcitabine by activating the c-MET/PI3K/AKT pathway [20]. Gemcitabine has been used in the treatment of metastatic CRC [13]; however, the mechanism of its anti-tumor activity and resistance remains largely unknown.

Consistently, in the present study, we observed that AGAP2-AS1 was overexpressed in CRC tissues and was strongly associated with advanced tumor stage and poor survival. Mechanically, we demonstrated that AGAP2-AS1 promoted the growth and metastasis, and induced gemcitabine resistance in CRC cells. We further found that AGAP2-AS1 exerted its tumor-promoting function through response to miR-497 and regulation of FGFR1.

LncRNAs usually exert their function via sponging miRNAs, which are important factors controlling tumor progression and development. MiR-497 is reported to have an inhibitory effect in multiple cancers, including non-small cell lung cancer (NSCLC), ovarian cancer, and pancreatic cancer [21–23]. Interestingly, our results showed that AGAP2-AS1 interacted with miR-497. To further explore the target gene of miR-497, FGFR1 was defined. FGFR1 is a member of the receptor tyrosine kinase (RTK) family, which triggers an intracellular signaling cascade that typically involves the MAPK and PI3K/Akt pathways [24]. The level of FGFR1 is found in many human cancers, including prostate cancer, lung cancer, and gastric cancer [25–27]. In addition, activation of FGFR1 promotes the development of epithelial-mesenchymal transition (EMT) in several human cancers [28]. In the present study, we found that AGAP2-AS1 regulated FGFR1 expression via interacting with miR-497. Moreover, overexpression of miR-487 or knockdown of FGFR1 abolished the promoting effects of AGAP2-AS1 on cell proliferation, migration and drug resistance. Therefore, our data revealed an oncogenic function of AGAP2-AS1 in CRC and AGAP2-AS1 exerts its function by regulating the miR-497/FGFR1 axis.

## CONCLUSIONS

In conclusion, our data demonstrated that AGAP2-AS1 is up-regulated in CRC specimens and associated with poor survival in patients, indicating that it may be a potential prognosis biomarker in CRC. Our study also revealed that AGAP2-AS1 is a novel oncogene for CRC, which functions through the miR-497/FGFR1 cascade.

## MATERIALS AND METHODS

### Patients and samples

A total of 116 pairs of human CRC tissues and matched adjacent normal tissues were retrospectively collected from the First Hospital of Jilin University. Informed consents were obtained from each patient. Patient clinical and pathological data were presented in Table 1. This study was approved by the Medical Ethics Committee of the First Hospital of Jilin University.

### Cell lines

CRC cell lines (DLD-1, SW480, HT29, CaCO2, RKO, HCT8) and 293T were obtained from the Cell Bank of the Chinese Academy of Science (Shanghai, China). These cells were preserved in Dulbecco's modified Eagle's medium (Gibco, NY, USA) supplemented with 10% fetal bovine serum (Gibco, NY, USA) and 100 U/ml penicillin and 100 μg/ml streptomycin. All cells were cultured at 37°C in an incubator with 5% CO_2_.

### qRT-PCR

The total RNA was isolated from tissues or cultured cells using Trizol reagent (Invitrogen) according to the instructions. For LncRNA and mRNA detection, complementary DNA was synthesized using the HiFiScript First Strand cDNA Synthesis Kit (CWBIO, China). qRT-PCR was carried out using the SYBR Premix Ex Taq II (TaKaRa, Dalian, China) on an ABI 7500 Real-Time System (Life Technologies, USA). GAPDH was used as an internal control. For miRNA detection, cDNA was synthesized using the TaqMan miRNA reverse transcription kit (Thermo Fisher Scientific, USA). U6 was used as an internal control.

### Western blotting

Cells were collected and lyzed in RIPA lysis buffer containing proteinase inhibitor. Total protein was separated by 10% SDS-polyacrylamide gel electrophoresis (SDS-PAGE) gel electrophoresis and then transferred to the (polyvinylidene fluoride) PVDF membrane (Millipore, MA, USA). The membrane was blocked with 5% free-fat milk and then incubated with anti-FGFR1 antibody (1:1000, Proteintech, USA) overnight at 4°C. After that, the membrane was incubated with Horseradish peroxidase-conjugated goat anti-mouse IgG second antibody (1:5000, Beyotime). Proteins were detected using Pierce ECL Western Blotting Substrate (Thermo Fisher Scientific, USA).

### Plasmids and siRNA interference

AGAP2-AS1 was synthesized by GenePharma (Shanghai, China) and inserted into the lentiviral vector pWPXL vector (GenePharma, Shanghai, China). AGAP2-AS1 fragments with the WT/mutant miR-497 binding sequence were constructed and cloned into pLuc vector. siRNAs for AGAP2-AS1 and FGFR1 were purchased from Thermo Fisher Scientific (Massachusetts, USA). siRNA transfection was performed using Lipofectamine 2000 reagent (Invitrogen, USA).

### Luciferase reporter assay

293T cells (1 × 10^5^ per milliliter) were cultured in 96-well plates and co-transfected with pLuc, pRLCMV, miR-497 mimics (NC) and pWPXL-AGAP2-AS1 using Lipofectamine 2000 reagent (Invitrogen, USA). The relative luciferase activity was evaluated using a dual luciferase reporter assay system (Beyotime, China) 48 h after transfection. All experiments were done in triplicate.

### Analysis of cell proliferation, migration, invasion and colony formation

As previously described [29], Cell Counting Kit 8 (CCK-8, Beyotime, China) and colony formation assay were used to measure the viability and clonogenic activity of CRC cells, respectively. Cross-well analysis was performed using a Boyden chamber (8 μm aperture, BD Biosciences) to measure the migration and invasion of CRC cells.

### Cell cycle and apoptosis analysis

Cell cycle and apoptosis analysis of AGAP2-AS1-highly expressed and silenced CRC cells was performed using flow cytometry with the cell cycle and apoptosis assay kit (CWBIO, China), respectively.

### RIP detection

RIP assays were performed using the Magna RIP kit (Millipore, Bedford, MA, USA) following the manufacturer's instructions. Cells were lysed in complete RIP lysis buffer and cell extracts were incubated in magnetic beads conjugated to human anti-ago2 antibody or negative control normal mouse IgG. The resulting RNA was then detected by qRT-PCR. U6 was used as a non-specific control.

### Tumor xenograft experiments

Twenty-four male BALB/c nude mice (aged 4 to 5 weeks) from Vital River Laboratory Technology (Beijing, China) were housed under sterile conditions and randomly divided into 4 groups: RKO/pWPXL, RKO/AGAP2-AS1, HT29/pWPXL, and HT29/AGAP2-AS1. 1×10^7^ RKO or HT29 cells stably expressing AGAP2-AS1 (RKO/AGAP2-AS1 or HT29/AGAP2-AS1) or bank vector (RKO/pWPXL or HT29/pWPXL) were subcutaneously injected into the flank of nude mice to form xenografts. Six weeks later, the mice were sacrificed and the weight of subcutaneous tumor was measured. All procedures were approved by the Animal Care and Use Committee of the First Hospital of Jilin University.

### Statistical analysis

Data were represented as mean ± SD. Data was analyzed by Student's *t* test, Mann-Whitney U test and x2 test. Survival rates were determined by Kaplan-Meier method. HR and 95% CI were calculated using the Cox proportional hazard model. The criterion of statistical significance was *P <* 0.05.
